# The use of global transcriptional analysis to reveal the biological and cellular events involved in distinct development phases of *Trichophyton rubrum *conidial germination

**DOI:** 10.1186/1471-2164-8-100

**Published:** 2007-04-11

**Authors:** Tao Liu, Qian Zhang, Lingling Wang, Lu Yu, Wenchuan Leng, Jian Yang, Lihong Chen, Junping Peng, Li Ma, Jie Dong, Xingye Xu, Ying Xue, Yafang Zhu, Wenliang Zhang, Li Yang, Weijun Li, Lilian Sun, Zhe Wan, Guohui Ding, Fudong Yu, Kang Tu, Ziliang Qian, Ruoyu Li, Yan Shen, Yixue Li, Qi Jin

**Affiliations:** 1 State Key Lab for Molecular Virology and Genetic Engineering, Institute for Viral Disease Control and Prevention, Chinese Center for Disease Control and Prevention. Beijing 100176, P. R. China; 2 Institute of Pathogen Biology, Chinese Academy of Medical Sciences, Beijing 100730, P. R. China; 3 College of Life Science, Jilin University, Changchun 130012, Jilin Province, P. R. China; 4 Research Center for Medical Mycology, Peking University, Beijing 100034, P. R. China; 5 Chinese National Human Genome Center_Beijing, Beijing 100176, P.R.China; 6 Bioinformatics Center Shanghai Institutes for Biological Sciences, Chinese Academy of Sciences, Shanghai, 200031, P. R. China

## Abstract

**Background:**

Conidia are considered to be the primary cause of infections by *Trichophyton rubrum*.

**Results:**

We have developed a cDNA microarray containing 10250 ESTs to monitor the transcriptional strategy of conidial germination. A total of 1561 genes that had their expression levels specially altered in the process were obtained and hierarchically clustered with respect to their expression profiles. By functional analysis, we provided a global view of an important biological system related to conidial germination, including characterization of the pattern of gene expression at sequential developmental phases, and changes of gene expression profiles corresponding to morphological transitions. We matched the EST sequences to GO terms in the *Saccharomyces *Genome Database (SGD). A number of homologues of *Saccharomyces cerevisiae *genes related to signalling pathways and some important cellular processes were found to be involved in *T. rubrum *germination. These genes and signalling pathways may play roles in distinct steps, such as activating conidial germination, maintenance of isotropic growth, establishment of cell polarity and morphological transitions.

**Conclusion:**

Our results may provide insights into molecular mechanisms of conidial germination at the cell level, and may enhance our understanding of regulation of gene expression related to the morphological construction of *T. rubrum*.

## Background

*Trichophyton rubrum *is a worldwide pathogen causing various superficial infections, accounting for at least 60% of dermatophytosis, such as tinea capitis, tinea corporis, tinea inguinalis, tinea manus, tinea unguium and tinea pedis [1-4]. This pathogen, which normally causes well-characterized superficial infections, also produces skin infections in unusual parts of the body in immunodepressed patients [5-7]. *T. rubrum *has a relatively simple and non-sexual stage life-cycle. Similar to numerous other filamentous fungi, it forms a mycelial colony *via *hyphal tip growth, branching and hyphal fusion. *T. rubrum *produces numerous asexual multinucleate spores, called macroconidia or arthroconidia, which are considered to be the primary cause of skin and nail infections in the host. The infection is triggered by the adherence of *T. rubrum *arthrospores upon contact with the status corneum (horny layer) of the skin. The spores then germinate and the mycelium forms. The longitudinal growth of hyphae can penetrate into the deep part of the horny layer, while breadthwise growth can aggravate skin damage [8-10].

Eukaryotic genomes contain various non-coding regions, including regulatory components, introns and repeat sequences, making these genomes much larger and more complicated than those of bacteria. In recent years, more and more eukaryotic genomes have been completed or assembled. *T. rubrum *had been shortlisted for sequencing by the Fungal Genome Initiative (FGD white paper, 2004), yet expressed sequence tag (EST)-based mRNA transcription profiling and microarray technology are used extensively to characterize gene functions and discover functionally related genes needed for developmental and behavioural processes [11,12]. *T. rubrum *consists of five chromosomes which range in size from 3.0 M bp to 5.8 M bp, and correspond to a total genome size at least 22.05 M bp [13]. We commenced the ESTs project of *T. rubrum *in 2002 and part of ESTs of the genes were obtained by our group in 2003 [14]. The project was completed recently. Extensive transcriptome data from eight life-cycle stages containing 11085 ESTs, which represent 80% of predicted genes, were obtained and annotated [15].

The prevalence of infections caused by *T. rubrum *and its human specific nature make it a good model for the study of human pathogenic filamentous fungi. The epidemiology, clinical case reports, strain relatedness, and drug susceptibilities of the organism are well documented [9]. However, little is known about its genetic and biological characteristics for *T. rubrum *that cause the most common fungal infection in humans [13]. In this study, we prepare a cDNA microarray consisting of 10250 ESTs to monitor the transcriptional pattern in a time serial process during *T. rubrum *germination. By functional analysis, we provide a global view of important biological system related to conidial germination. In addition, we hypothesize that the expression levels of genes related to some fundamental biological events, such as germination process, protein synthesis and other metabolic pathways activation, may be involved in the distinct steps of *T. rubrum *germination and sequential morphological transitions by impacting on distinguished cellular processes. Since signal transduction pathways and cell processes have been elucidated in the budding *S. cerevisae*, establishing a morphogenetic network that links cell-cycle events with cellular morphogenesis, we matched the ESTs sequences to the *Saccharomyces *Genome Database (SGD) and a number of homologues of *S. cerevisae *genes related to signalling transduction pathways and different cellular events, such as response to environmental conditions, maintenance of cell wall integrity and establishment of cell polarity were found to be involved in *T. rubrum *conidial germination. Our results at the cell level may provide stepping stones on the way to further study of the molecular mechanisms of *T. rubrum *conidial germination, and may enhance our understanding of some similarities between the morphogenetic networks of yeast and the filamentous fungi. Since it has been proposed that the identification and targeting of conidial germination-specific processes provides an excellent strategy for drug and fungicide development in these pathogenic fungi [16], this study may provide some clues for further approaches to finding new drug targets.

## Results

### Morphological transitions during conidial germination

**Figure 1:**
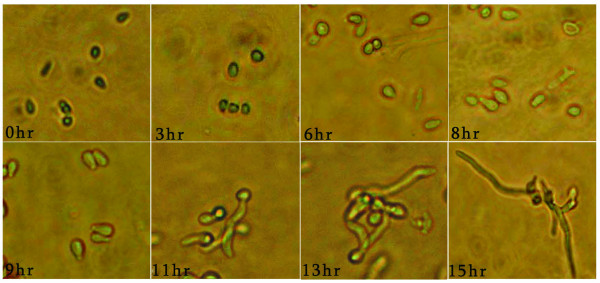
**Morphological transitions during conidial germination of *T. rubrum***. Dormant conidia were introduced into Sabouraud liquid medium and incubated at 28°C. 0 hr is dormant conidia. The 0 hr to 15 hr images were taken at the indicated time-points, and show the morphological transitions with time.

In *T. rubrum*, there are two visible morphological transitions in the course of germination. The first morphological transition occurred at 3–4 hr after incubation. In this stage, the conidia appear swollen and bright under microscopy. The second transition occurred at 9–10 hr after incubation. Most conidia budded and the germ tube emerged at the end of the conidia in this stage. The distinct morphological states in germination are shown in Figure 1.

### Prominent transcriptional changes during T. rubrum conidial germination

In order to monitor the transcriptional changes that occur during *T. rubrum *conidial germination, we assembled an expression microarray that consists of 10250 ESTs. Using the microarray, the abundance of mRNA in samples collected at 1 hr intervals throughout the 15 hr time-course of conidial germination was evaluated. In the test procedure, we use genomic DNA as the reference control to normalize the relative expression levels under the mRNA hybridization signals. The genomic normalization procedure provided reproducible hybridization signals for 96% of the 10250 ESTs.

**Figure 2:**
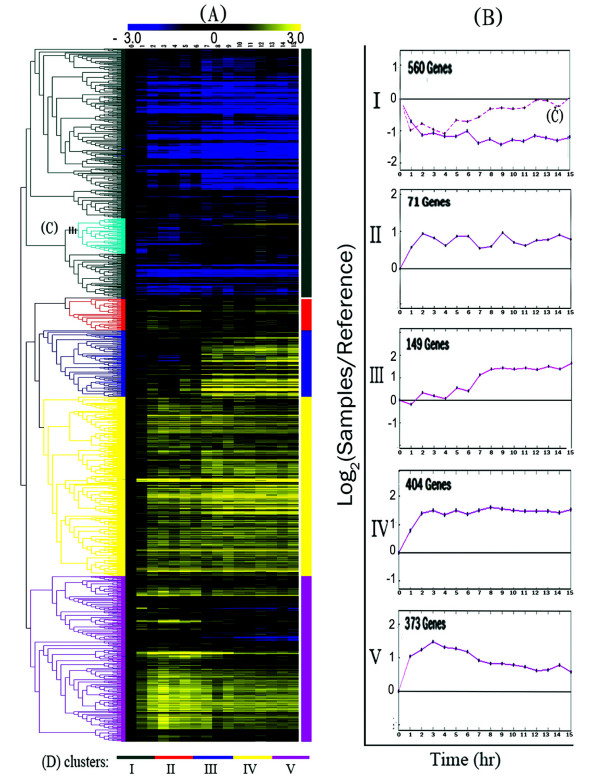
**Hierarchical clustering of microarray data and identification of genes with similar transcriptional profiles**. (A) Total of 1561 genes was clustered on the basis of their expression profiles across the sixteen time-points using TIGR MultiExperiment Viewer (MeV) software[17]. Each gene's expression values were standardized to have a median of zero and a standard deviation of one across the sixteen time points. The lighter colour in the cluster dendrogram is correlated with a higher expression level. Five distinct clusters were selected visually, and the node separating each cluster is shown in the distance tree. (B) An average expression profile of genes within each cluster. To obtain each profile, a sum of expression values across the sixteen time-points for each gene was standardized to 1. Next, time-course values for all genes in each cluster were summed, and the summed value for the sixteen time-points for each cluster was scaled to 1. (C) A sub-cluster of cluster I. (D) The name of each cluster and representative profile is shown in deferent colour.

One-thousand five hundred seventy six putative genes which expression levels strongly changed during germination were selected to identify the correlation between expression profile and the events that occurred during germination. The 1576 genes were subjected to hierarchical clustering analysis using TIGR MultiExperiment Viewer (MeV) software [17]. Five representative clusters were chosen visually, each of which showed distinctive profiles (Figure 2). The genes enrichment within cluster and the statistical significance of gene distribution across the clusters were estimated by post-hoc tests (detailed results see Additional files 1). The majority of genes (1561 out of the total of 1576 genes) belonged to one of the five chosen clusters. In order to verify the microarray result, the relative expression levels of 8 genes at several time points (0, 4, 10, and 15 hr) were estimated by Quantitative real-time RT-PCR. The results showed a strong positive correlation between the two techniques (detailed results see Additional file 1).

Cluster I contains 560 genes that showed maximum expression levels in dormant conidia, most of them with expression levels dropping quickly during conidial germination. A sub-cluster containing 80 genes shared a lowest expression at 4 hr but was induced at subsequent time-points. These data indicated that cluster I mRNAs are present in dormant conidia before the initiation of germination and must be stored in conidia before encapsulation. The 71 genes contained in cluster II were obviously induced during 0–2 hr. Their average expression levels were lower than those of genes in cluster IV and fluctuated several times throughout the process of germination. Cluster III contains 153 genes that had low and constant expression levels until 4 hr, but had increased expression levels subsequently. Cluster IV contains 404 genes and cluster V contains 373 genes. Transcripts of the genes in these two clusters were induced to increase from the beginning of germination. The genes in cluster V with a peak expression at 3–4 hr showed decreased levels of expression at the succeeding time-points, whereas the expression levels of genes within cluster IV were increased significantly during the first 3 hr but showed a higher or relatively constant trend thereafter.

### Analysis of expression profiling associated with putative biological and physiological events during *T. rubrum *conidial germination

To identify the correlation between gene expression profile and physiological transitions during germination, ESTs of the selected 1576 genes were matched to the sequences in the Gene Ontology (GO) database by BLASTX searches[18,19]. The GO identifiers for each target were determined on the basis of the BLASTX score and the GO evidence code (Table 1, for detailed results, see Additional file 2).

As mRNA was stored in conidia, the expression levels of genes in cluster I declined from the onset of germination. Function annotation showed that about 70 genes out of 1561 were predicted with function related to "response to external or abiotic stimulus" and 30 of those fall into cluster I. These results were consistent with the expectation that genes in cluster I may be involved in perceiving the changes of environmental conditions and play important roles in triggering conidial germination. Cluster I had 15 of 29 genes assigned the function "transcription" and the largest group assigned the function "regulation of biological process" and "cell communication" containing about half of the two categories of genes. The results implied that the transition from the dormant state to vegetative growth was a very complicated process, and needed various regulation pathways to respond to the changes of environmental conditions and initiate transcription to induce germination.

There are 15 genes devoted to "development" and 12 of them are in cluster I. Similar results were reported Qikai Xu for spore germination in *Dictyostelium discoidium*, who suggested that a major event in germination is the erasure of the developmental state [20]. We suppose that those genes may be involved in maintaining the dormant state. Expression levels of a sub-cluster of genes in cluster I increased after a decrease from 0 to 4 hr. These genes may be stored in the conidia and required for later growth during germination.

When germination is induced, the conidia initiate isotropic growth. Morphologic investigation showed that the isotropic growth phase continued from 0 hr to 9 hr. In this phase, conidia became brighter and the diameter was larger than that in the dormant phase. Genes in cluster II, IV and V were induced and increased their expression levels immediately following the onset of germination. The difference between genes in these three clusters was that the expression level of cluster V genes was highest at 3–4 hr and decreased thereafter, whereas those in clusters II and IV increased continuously. This result implies that genes in all three clusters are involved in isotropic growth but only those in clusters II and IV were needed for germ tube emergence and vegetative growth.

Function annotation indicated that 401 out of all 1561 genes function in "metabolism". The largest group of 137 of those genes belonged to cluster V, 17 belonged to cluster II and 95 belonged to cluster IV. Apart from 117 of the genes in cluster I and 35 genes in cluster III, the expression levels of most metabolism-related genes increased immediately at the onset of germination and approached the highest expression levels.

About 149 of all 1561 genes functioned in "protein synthesis" and 88 of those are in cluster V, 5 are in cluster II and 17 are in cluster IV. This is consistent with the results that had been obtained for *A. nidulans*: cloning of genes that encode ribosomal proteins (*rp *genes) was shown to be induced rapidly upon activation of spore germination, and their mRNA levels were lower than those in the subsequent polarized growth [21]. In *N. crassa*, previous work had showed that a group of genes that function in protein synthesis and as ribosome components were over-represented and had maximum expression levels in the first 4 hr after induction of germination [22]. A total of 173 of all 1561 genes predicted in "biosynthesis" have the expression profiling similar to that in "protein synthesis".

Genes in cluster III were induced at 6 hr and may not be required in the early stage of germination but are involved in polarity growth and germ tube formation. Most genes involved in biological process in cluster III are devoted to "metabolism". Gene expression profiling analysis showed that genes in cluster II and IV also have high expression levels and may be involved in later phase of germination. In polarity growth phase, a large group of genes function in "protein synthesis" belongs to cluster V, which expression levels were fallen from 4 hr during germination. However more than half of genes related to "catabolism" belonged to cluster III and IV, their expression levels were increased as time extended. These results suggest that catabolism may become more active at polarity growth stage.

### Comparison of signalling transduction pathways and cellular events involved in conidial germination in *T. rubrum *and yeast

To better understand the cellular processes and signalling pathways involved in conidial germination, the EST sequences of all 1561 genes were matched to GO terms at the *Saccharomyces *Genome Database (for detailed results, see Additional file 2). A total of 37 homologues of genes involved in several signalling pathways or regulation modules, such as the cAMP/PKA signalling pathway, the Ras-GTPase-related signalling pathway, the MAPK signalling pathways and two-component signal transduction systems were found and their expression levels were induced during the process (Table 2, more detailed results are also provided in Additional file 3). These results suggest that these signalling modulus may be involved in regulation of germination of *T. rubrum*.

cAMP/PKA signalling pathways and RAS-like GTPase signalling have been characterized, though are not always equivalent in function, playing important roles to perceive the external signal and initiate the germination in *S. cerevisae*, *A. nidulans *and other fungi [23,24]. To *T. rubrum*, a *sch9 *homologue, DW685958, was found, and it belongs to cluster I. *sch9 *is *sch9A *in *A. nidulans *and seems to be involved in cAMP-independent activation of a PKA target in response to nitrogen or amino acids when a fermentable carbon source is abundant [23-27], and is important in spore germination in a.*nidulans *and in *S. cerevisiae*. Three Ras2 homologues, DW680378, DW683586, and DW679797, were identified during conidial germination of *T. rubrum*. DW683586/*ras2 *is in cluster I, and DW680378/*ras2 *and DW679797/*ras2 *are fallen in cluster V. These results suggest that Ras-related GTPase and cAMP/PKA signalling pathways may also play roles in of conidia germination in *T. rubrum*.

Three homologues of S. *cerevisae *genes *sln1*, *ypd1 *and *skn7 *related to two-component signal transduction systems were found, and DW695308 and DW705117 belong to cluster I. Sln1p regulates the HOG1 mitogen-activated protein kinase (MAPK) in response to changes in extracellular osmolarity in *S. cerevisae *[28]. Sln1p also mediates a phosphorelay to a second response regulator, Skn7p, in the cell [29]. Since two-component signal transduction systems are very conservative, they may have similar effects in conidial germination of *T. rubrum*.

In order to characterize the regulation systems involved in different phases during germination, the homologue genes of *S. cerevisae *found in all 1561 genes were also compared with the genes in "cell wall" and "polarity" related GO terms at *Saccharomyces *Genome Database (Table 3 and 4, more detailed results are also provided in Additional files 4 and 5,6 respectively).

There are 20 homologues of *S. cerevisae *genes related to "cell wall" induced during conidial germination and 15 of them fall in clusters II, IV and V (Table 3). These genes had a high level of expression in the isotropic growth phase. These results are consistent with the expectation that "cell wall"-related cellular processes were induced to avoid the increased volume resulting in cell lysis/bursting in isotropic growth [21]. A total of 10 out of those 20 homologue genes were characterized as being related to the Rho-pkc-MAPK pathway, the MAPK signalling pathways or the septation machinery (related to the P-loop GTPases), or their interacting proteins, which indicates that these signalling pathways may be involved in the regulation of isotropic growth.

The isotropic growth phase finally leads to polarity growth and results in the formation of a germ cell in both yeast and filamentous fungi [30]. There are 15 homologues of *S. cerevisae *genes devoted to the development of cell polarity found in the germination process and 14 of those belonging to cluster II, III and IV, respectively, having maximal expression levels in the polarity growth stage (Table 4). Out of those genes, 9 of their homologue genes in *S. cerevisae *are related to the Rho-GTPase, Ras-GTPase, MAPK and cAMP/PKA signalling pathways, and the septation machinery or their interacting proteins; 8 of them are in cluster IV or cluster III and have increased expression levels in polarity growth. Most of these genes are related to Rho-type GTPase modules, septation machinery and the MAPK signalling pathways. These results suggest that the Rho-type GTPase modules, the septation machinery and MAPK signalling pathways may play roles in regulation of cell polarity development during conidial germination in *T. rubrum*.

## Discussion and Conclusion

As in other filamentous fungi, the conidial germination process in *T. rubrum *can be divided into three steps: (1) an activation step triggered by environmental factors; (2) an isotropic growth phase representing the first morphological event, referred to as swelling; and (3) a polarized growth phase. In *T. rubrum*, conidial germination is accompanied by two morphological transitions, swelling and germ tube emergence. The swelling appears at 3–4 hr and continues until germ tube emergence at 9–10 hr after induction of germination. In order to monitor the correlation between the pattern of gene expression with the biological and physiological events that occur during germination, a cDNA microarray containing 10250 ESTs was developed and the gene expression profiling was evaluated in a 15 hr time-series experiment throughout the process of germination.

Traditional protocols for microarray data normalization use a 'control' RNA sample from a particular tissue or time point (RNA normalization), a pool of 'grouped' RNA samples from different tissues or different time points. There are several problems with these approaches. For example, at some growth conditions or time point, the transcription levels of some genes will be undetectable (or very low). Furthermore, for microbial systems, the 'grouped RNA normalization' procedure may require pooling RNA from 20 or 30 experimental conditions at different growth phases. In response to these problems, genomic DNA standards for gene expression profiling had been tested and proposed by Talaat *et al *[31]. In this report, we used genomic DNA as the reference control to normalize the relative expression levels under the mRNA hybridization signals. The genomic normalization procedure provided reproducible hybridization signals for 96% of the 10250 ESTs. Our results suggest that genomic DNA standards can also expediently be used to evaluate the gene expression profiling in *T. rubrum*.

A total of 1561 genes with strong statistical estimates on expression levels were obtained and clustered. The results show that the alteration of gene expression levels does not correspond to morphological transitions at certain time-points. There are three turning points, at 0 hr, 2–3 hr and 6–7 hr, on the curves of gene expression profiling during germination. Gene clustering and function analysis suggested that isotropic growth is induced immediately after the initiation of germination, and polarity growth may start at 6 hr or earlier during germination. These results imply that changes of genes expression profiling precede the morphological transitions.

The ability of fungal spores to store pre-packaged mRNA has been revealed in *S. cerevisae*, *A. nidulans *and *N. crassa *[32,33]. These stored mRNA are primed for rapid activation and translation in the presence of nutrients. The decay of spores containing mRNA is induced soon after germination [34]. In this study, mRNA of genes in cluster I existed in spores before induction of germination and the expression levels of those genes were down-regulated immediately at the onset of germination. Functional annotation showed that more than half of the genes related to "transcription", "cell communication" and "regulation of biological process" are in cluster I, and there are many genes in this cluster that function in "response to external or abiotic stimulus". These results imply that stored mRNA is important for the activation of germination and the transition of spores from the dormant state to vegetative growth is complicated, in that it is induced and regulated by both exterior factors and intra-cell signalling.

Spores are not simply quiescent cells, a basal level of RNA and protein synthesis is required for spore survival. So, we supposed that cluster I may include two types of genes. One type may be stored in conidia and induce decay after translation during germination, whereas the second type may have specific expression in conidia that decayed immediately after induction of germination. Most genes that function in "development" are in cluster I and are down-regulated at the onset of germination. Similar results have been reported by Xu *et al *[20]. We suppose that genes may be involved in maintaining the dormant state. Since we have little knowledge about the events that occur in dormant conidia and the activation of germination in *T. rubrum*, the identity of the spore-contained mRNA may provide some clues for further research on the molecular mechanism of conidial survival and germination in *T. rubrum*.

The expression levels of genes in clusters II, IV and V that increased rapidly after induction of germination may be involved in isotropic growth. Gene functions in "metabolism" also are immediately induced and have high expression levels in isotropic growth indicate that dormant conidia breaking dormant state is a very quick process.

Protein synthesis has been shown to be required for germination [35-37]. In our data, most genes function in "protein biosynthesis" belongs to those clusters and achieve the maximum expression levels in isotropic growth. These results are consistent with previous reports that conidia contain fully active ribosomes and it is therefore likely that the activation of the *rp *genes during conidial germination, and hence *de novo *synthesis of ribosomes, is a prerequisite to achieve a growth rate appropriate for germ tube emergence that could not be obtained by the sole use of the translation machinery stored in the conidia [21]. With the passage of time, transcription of the genes related to "catabolism" was induced and became more active, and the conidia gradually enter the vegetative growth state.

The budding yeast *S. cerevisiae*, with the powerful tools applicable in this organism, offers the opportunity to rapidly characterize signal transduction pathways of a eukaryotic cell in great detail. Therefore, yeast may be used as a reference library-much as its genome sequence has been-for the analysis of conserved signaling pathways, e.g., in the more complex, multicellular filamentous fungi [38]. To better understand the mechanism and cellular events be involved in *T. rubrum *conidial germination, we matched The ESTs of the selected 1561 genes to GO terms at the *Saccharomyces *Genome Database. Some homologues to *S. cerevisae *genes related to signalling modules and important cellular processes were found and distributed in various clusters. Our results indicated that though the molecular mechanism and biological processes are differ in morphological construction, there are several conservative signalling pathways and similar cellular events between budding yeast and *T. rubrum *germination.

The primary requirement for initiation of germination and completion of the subsequent steps is the sensing of external signals. In *S. cerevisae *and *A. nidulans*, glucose or other fermentable carbon sources as external signals are necessary and sufficient to trigger spore germination. In *S. cerevisiae*, glucose sensing is mediated by the G-protein-coupled receptor (GPCR) Gpr1p that in turn activates the heterotrimeric G-protein α-subunit encoded by the *GPA2 *gene [39,40]. The Gpr1p-Gpa2p system mediates glucose-dependent activation of the cAMP-dependent protein kinase (PKA) pathway that is associated with mobilization of trehalose, decreased stress resistance, and expression of ribosomal protein (*rp*) genes [39]. The Ras pathway is the rate-limiting step of spore germination, since an elevated level of activated Ras protein increased the rate of germination [37,41]. In *A. nidulans*, a very recent report has revealed GanB regulates conidial germination within the heterotrimeric G-protein GanB(α)-SfaD(β)-GpgA(γ) through activation of the cAMP/PKA pathway in response to glucose. A previous study revealed that RasA from *A. nidulans *regulates conidial germination via an undefined signalling pathway in parallel to the cAMP/PKA pathway. cAMP/PKA signalling controls early events of conidial germination in response to sensing a carbon source, and plays a critical, but not essential, role in the germination process. Indeed, inactivation of adenylate cyclase results in a severe delay in, but not a complete arrest of, germ tube emergence. Ras signalling may be a response to carbon source signalling initiating germination, but plays more important roles in inducing conidial germination [23]. In our data, genes in cluster I are present as mRNA in the conidia and may respond to external signals triggering the germination. Several homologues of genes related to the cAMP/PKA and RAS GTPase signalling pathways were found related to conidial germination in *T. rubrum*. However, we also found that glucose only could not trigger the conidia germination and conidia could germinate and grow in nitrogen abundant but fermentable C source absent media such as soy protein liquid medium (SP) and keratin liquid medium (KSP) [42-44]. So, we suppose that the signal transduction modules are different molecular mechanism involved in sensing the external signal and initiation of germination in *T. rubrum*.

Isotropic growth results in a drastically increased volume. In our data, the "cell wall"-related process genes were significantly induced and most of them had the maximum expression levels in the isotropic growth phase. These results indicate that cell wall biosynthesis and integrity maintenance are also very important for isotropic growth of *T. rubrum*. Cell wall biosynthesis and monitoring of cell wall integrity is important to avoid cell lysis/bursting and may account for the increase of gene expression levels related to "cell wall". Two genes related to the MAPK and the Ras/Rho-type GTPases signalling pathways were found and shown to have similar expression profiling. Since Rho-GTPases and the MAPK signalling pathways had been characterized as being involved in the regulation of cell-wall biosynthesis in *S. cerevisae *and some other filamentous fungi, these signal transduction modules may play roles in cell-wall biosynthesis and be involved in the regulation of isotropic growth.

Polarity growth is also essential for germination and hyphal growth. In *Ashbya gossypii*, *Agbem2 *mutant loss of cell polarity eventually caused completely isotropic growth, resulting in large, balloon-shaped tip cells [30]. Comparison with the GO terms at the *Saccharomyces *Genome Database indicated that there are 23 homologues of *S. cerevisae *genes devoted to "cell polarity development" found be involved in the germination process and half of those share high expression levels at polarity growth phase. Our data also suppose that Rho-type GTPase modules, septation machinery and MAPK signalling pathways may take part in regulation of cell polarity development and play roles in conidial germination of *T. rubrum*.

Two-component signal transduction pathways were found that may be involved in the conidial germination of *T. rubrum*. Two-component signal transduction pathways are used extensively to mediate prokaryotic signalling events. In recent years, these signalling systems have been found in eukaryotes, including plants, yeasts, filamentous fungi and slime moulds [45,46]. In *C. albicans*, these signal transduction pathways regulate cell-wall biosynthesis (and, therefore, adherence to host cells), osmotic and oxidant adaptation, white-opaque switching, morphogenesis, and virulence of the organism [47]. Since the presence of two-component signal transduction pathways has not been demonstrated in mammals, further study on these signalling pathways may lead to the discovery of new drug targets for *T. rubrum *control.

## Methods

### Strain and culture conditions

*T*. *rubrum *strain BMU01672 was isolated from nail scrapes of a patient suffering from tinea unguium. The strain was confirmed as *T. rubrum *by morphologic identification, as well as by PCR amplification and sequencing of the 18 S ribosomal DNA and internal transcribed spacer (ITS) regions. Strain reference samples are stored at the Research Centre for Medical Mycology, Beijing, China.

*T. rubrum *was grown on potato glucose agar (Difco) at 28°C for 10 days to produce conidia [48]. The conidia were washed from the medium at 4°C with distilled water and passed through a 70 μm pore size nylon filter twice to remove hyphal fragments. The conidia sample was centrifuged at 1800 g for 10 min, and then resuspended to give a final concentration of 10^8 ^conidia/mL.

For germination analysis, 20 mL of conidia suspension was introduced into each of a series of 250-mL flasks containing 80 mL of Sabouraud liquid medium (containing 49 g of glucose, and 10 g of Difco Bacto peptone in 1 L of distilled water), and immediately incubated at 28°C with constant shaking (200 rpm, Innova 4230 Refrigerated Incubator Shaker, New Brunswick Scientific, Edison NJ). Two samples at time-point 0 hr were taken directly from the stored conidia. Subsequent samples were harvested every hour from the first to the fifteenth hour during the germination process. Two samples were collected from the flasks as independent biological duplicates, so a total of 32 samples were obtained during the whole process of germination from 0 hr to 15 hr

### Microscopy

Morphological transitions during germination were examined using an OLYMPUS CKX41SF microscope (OLYMPUS Optical CO., LTD), and viewed under a 40 × /0.55 PH2 objective lens. Micrographs were taken with an OLYMPUS digital camera and captured images were processed using Adobe Photoshop software.

### cDNA microarray construction

PCR fragments used for printing the microarray chip were amplified from the EST library in 96-well plates using vector-PCR amplification with T7 and SP6 universal primers on the GeneAmp PCR system 9600 (Perkin Elmer, Foster City, CA). A single EST cDNA clone was selected to represent each assembled sequence (i.e. putatively unique transcript). For an assembled contig represented by multiple ESTs, the rule followed was to select the clone with the most extensive DNA sequencing read length. PCRs were initiated with DNA denaturation (5 min at 96°C) followed by annealing for 30 s at 55°C and extension (1 min and 30 s at 72°C) steps carried out for 35 cycles. PCR products were puried using the QIAquick PCR Puri^®^cation kit (Qiagen) and eluted in 100 μl sterile water. Purified PCR products were dried and then resuspended with a concentration of 100 ng/μL.

A set of microarrays containing a total of 11,232 spots, including 10, 250 clones in the form of PCR products and 982 controls in each block, including blank controls, negative controls, and positive controls, were spotted onto CMT-GAPSII-coated slides (Corning Glass) using a 16-pin configured MicroGrid II array printer (BioRobotics) controlled by the MicroGrid TAS Application Suite (version 2.2.0.6).

The spotted cDNA was cross-linked to the surface of the slides (at 65 mJ) by using a StrataLinker instrument and washed with a 1% SDS solution to minimize the background. Slides were subsequently placed in a blocking solution containing 200 mM succinic anhydride and 50 mM sodium borate prepared in 1-methyl-2-pyrrolidinone for 20 min, washed for 2 min in 95 °C distilled water, and rinsed five times in 95% ethanol. Slides were spin dried at 500 rpm for 5 min and stored for future hybridizations.

### RNA extraction and cDNA labelling

Samples were centrifuged at 1800 g for 10 min at 4°C and immediately grounded into mortars with liquid nitrogen. Total RNA were isolated and purified following the protocols of the RNeasy^® ^Plant Mini Kit (Qiagen, Valencia, CA). The RNA concentration and purity were determined spectrophotometrically by measuring absorbance at 230, 260, 280, and 320 nm. The purity and integrity of the RNA samples were confirmed by agarose gel electrophoresis. Poly (A)+ mRNA was isolated with Oligotex mRNA Mini Kit (Qiagen).

Test mRNA enriched from 50 μg of total RNA was reverse transcribed into cDNA and labeled with a fluorescent dye (Cy3 or Cy5). Also, 5 μg of reference genome DNA isolated in the hyphal stage was labelled. Test samples and reference samples were dye-swapped to avoid variation arising from the dyes.

### cDNA microarray hybridization

Labelled cDNA and reference genomic DNA were purified using the QIAquick PCR Puri^®^cation kit (Qiagen), then mixed and resuspended in 10 μL of distilled water, mixed with 1.5 μL of 50 × Denhardts, 2.25 μLof 20 × SSC, 1.125 μL of 500 μg/mL yeast tRNA, 0.375 μL of 1 M HEPES (pH 7.0), 0.375 μL of 10% SDS, 2 μL of poly (A) (5 mg/μL). The mixture was heated 100°C for 3 min. Hybridization was carried out as described by Hayes *et al *[49].

### Image acquisition and data analysis

The processed slides were scanned with a GenePix 4100B scanner (Axon). Fluorescent spots and the local background intensities were quantified with GenePix Pro 6.0 software (Axon). The local background value was subtracted from the intensity of each spot. The mean of the signal intensities of the control spots hybridized with labeled reference genomic DNA in each experiment was calculated. Ten different *A. thaliana *genes and human β- actin gene from SpotReport™ cDNA Array Validation System (Stratagene) were used for the controls. The spots that showed intensity with labeled reference genomic DNA that was lower than the mean value of the control spots were excluded from further analysis. The mean log2 (sample/reference) ratios of signal intensity were calculated for analysis [50].

The raw data consisted of 64 hybridizations (32 dye-swap pairs) on duplicate printed slides from the same batch. At every time-point, two biological replicates containing 4 hybridized data were used for further analysis. These multiple data sets were flagged, fitting all the features: spot Dia. > = 80 μm, %B (532 or 635) + 2SD > 55, SNR635 (or 532) > = 3 and normalized (the ratio of medians of all features equal to 1) by the GenePix Pro software (version 6.0). The data sets were further normalized in two steps: total intensity and Lowess normalization using Tiger MIDAS V2.19 [17].

After spots flag and normalization process, 9470 spots (about 93.7% of all) in a microarray were used for the gene expression profiling analysis. The expression variations during the time-course were analyzed by ANOVA (*p *< 0.01) for each gene, using Tiger TMEV 3.1 software [17]. 2772 genes were obtained that their expression levels were significantly altered during the germination. In order to identify genes whose expression levels were altered dramatically during germination, 1576 out of the 2772 genes fitting expression levels altered exceeding twofold were used for further analysis. The estimated expression levels of these genes were clustered by Tiger TMEV 3.1 software using a hierarchical clustering method, in which similarity in expression patterns between genes is measured as Pearson's correlation coefficient, and the closest two genes or clusters are successively joined. Distances between clusters represent the average distances between genes in the clusters. Five representative clusters were chosen visually, each of which showed distinctive profiles (Figure 2). The genes enrichment within cluster and the statistical significance of gene distribution across the clusters were estimated by post-hoc tests(*p *< 0.05) using SPSS 10.0 For Windows (detailed results see Additional file 1) [51].

To annotate the putative functions of selected genes and predicate the biological process involved in germination, we used a controlled vocabulary for describing gene function [18,19]. The ESTs of 1576 selected genes were matched to the sequences in the GO database. GO identifiers were determined on the basis of the BLASTX score and the GO evidence code of the homologous genes (see Additional file 2 for details).

### Quantitative real-time RT-PCR

In order to verify the microarray result, the relative expression levels of 8 genes at several time points (0, 4, 10, and 15) were estimated by Quantitative real-time RT-PCR. First-strand cDNAs were synthesized from 2 μg of total RNA in a 100-μl reaction volume using the SuperScript First-Strand Synthesis System for RT-PCR (Invitrogen, Carlsbad, CA) in accordance with the manufacturer's instructions. Quantitative real-time PCR experiments were performed in triplicate using the 7000 Sequence Detection System (Applied Biosystems, Foster City, CA). Independent PCRs were performed using the same cDNA for both the selected genes and the 18S rRNA, using the SYBR Green PCR Master Mix (Applied Biosystems). Gene-specific primers were designed for the genes and 18S rRNA using Primer Express software (Applied Biosystems) and are shown in Additional file 1. The PCR cycle consisted of AmpliTaq Gold activation at 95°C for 10 min, followed by 40 cycles of denaturation at 95°C for 15 s and annealing/extension at 58°C for 1 min. A dissociation curve was generated at the end of each PCR cycle to verify that a single product was amplified using software provided with the 7000 Sequence Detection System. The changes in fluorescence of SYBR Green I dye in each cycle were monitored by the system software, and the calculated threshold cycle (*C*_t_) for each gene amplification was normalized to *C*_t _of the 18S rRNA gene amplified from the corresponding sample before calculating the fold change from a selected time point to 0 time point using the following formula:

fold change = 2^ΔΔ*C*^_t_

where ΔΔ*C*_t _for gene *j *= (*C*_t,*j *_- *C*_t,18S rRNA_)_a time point _- (*C*_t,*j *_- *C*_t,18S rRNA_)_time point 0_.

## Accession Numbers

EST sequences used for cDNA microarray preparation were deposited in GenBank under accession numbers: [DW405580–DW407270 and DW678211–DW711189]. The microarray related data were submitted to Gene Expression Omnibus (GEO) under accession number: [GSE5083]

## Authors' contributions

TL carried out the *T. rubrum *functional genomics studies, EST sequence analysis, cDNA microarray preparation, the gene expression analysis and participated in drafting the manuscript. QZ contributed to cDNA microarray preparation and the gene expression analysis. LW, LY, WL, JP, JY, LM contributed to EST sequencing, data analysis and cDNA microarray preparation. LC, JD, XX, YX, WZ, YZ, LY, LQ, DY, GD, KT participated in EST sequence analysis. LS, WL, ZW contributed to *T. rubrum *material preparation. RL, YxL, YS participated in design of the studs. QJ proposed the research goal, supervised the whole studies and provided a critical review of the manuscript. All authors read and approved the final manuscript.

## Supplementary Material

Additional file 1 The data provided statistical analysis results for verifying the statistical significance between clusters of selected genes and real time PCR verification for microarray data. Click here for file

Additional file 2 The data provided microarray data and the results of the selected 1562 genes matching to GO and SGD. Click here for file

Additional file 3 The file provided more detailed results of Table 2. Click here for file

Additional file 4 The file provided more detailed results of Table 3. Click here for file

Additional file 5 The file provided more detailed results of Table 4. Click here for file

Additional file 6 The file provided GO matching results and relative biological process terms of the selected genes in the paper. Click here for file

## Figures and Tables

**Table 1:** Putative predicated function and biological processes of selected genes

GO term*	**Gene number**	**Clusters**				
Biological process	**719**	**I**	**II**	**II**	**IV**	**V**
Metabolism	401	117	95	137	35	17
Biosynthesis	173	26	6	13	38	90
Catabolism	30	9	0	8	8	5
Amino	36	17	4	4	9	2
Lipid	20	4	0	5	8	3
Energy pathways	28	7	0	1	15	5
Carbohydr.	33	12	0	5	9	7
Protein biosynthesis	149	30	5	9	17	88
Nucleic acid	97	38	4	3	32	20
Transcription	29	15	1	1	5	7
Response to stimulus	70	30	7	5	13	15
Regulation of biological process	34	18	2	0	6	7
Cell communication	30	13	0	4	4	9
Development	15	12	1	0	2	0
Reproduction	12	4	0	4	2	2
Transport	73	21	1	6	25	20
Cell organization and biogenesis	28	12	0	3	8	10
Cell adhesion	11	3	0	5	2	1
Biological process unknown	113					

* ESTs of the selected genes were matched to the sequences in the Gene Ontology. Total 780 out of 1561 genes were assigned to different biological process, and 666 and 960 out of 1561 genes were assigned to GO terms: "Cell Components" and "Molecular Function" respectively. Individual gene can have multiple Gene Ontology assignments. About 454 genes have no hints in the Gene Ontology. The detailed results are provided in the Additional file 2 and 6.

**Table 2:** Putative genes important for signal transduction during *T. rubrum *conidia germination

**Signaling modules**	***S. cerevisae *Genes**	***T. rubrum *homolgues**	**E-value**	**Clusters**
**cAmp-dependent protein kinase inhibitor activity**				
	GIS2	DW703096	2.00E-31	V
	SCH9	DW685958	1E-10	I
**MAP kinase activity**				
	BCY1	DW704384	1.00E-66	V
	SFA1	DW699725	5.00E-50	III
	HSP12	DW689141	4.00E-11	I
	SLT2	DW703091	1.00E-112	IV
	PTP2	DW682511	2.00E-13	I
	RDI1	DW708650	9.00E-44	III
	HRT1	DW710468	6.00E-38	IV
	HXT6	DW694189	6.00E-20	I
**GTPase and GTPase related signaling**				
	CDC10	DW698270	2.00E-17	IV
	CDC10	DW693198	2.00E-17	IV
	DDR48	DW704176	3.00E-43	III
	DDR48	DW679616	3.00E-43	I
	DDR48	DW680636	3.00E-43	I
	LSG1	DW685586	3.00E-53	I
	RAS2	DW680378	2.00E-24	V
	TUF1	DW702910	8.00E-41	V
	YPT52	DW682488	4.00E-26	I
	GSP1	DW680214	5.00E-48	I
	YRB1	DW698798	1.00E-13	IV
	YRB1	DW681957	1.00E-13	III
	SIT1	DW688840	2.00E-94	I
	GAC1	DW692202	3.00E-10	I
	HSL1	DW696303	1.00E-09	III
	RDI1	DW708650	9.00E-44	III
	GDI1	DW704231	8.00E-91	III
	HRT1	DW710468	6.00E-38	IV
	GIS2	DW703096	2.00E-31	V
	RAS2	DW679797	2.00E-24	V
	RAS2	DW683586	5.00E-16	I
**two-component regulatory systems**				
	YPD1	DW695308	7.00E-15	I
	SKN7	DW705117	5.00E-11	I
	SLN1	DW684321	5.00E-07	V

**Table 3:** Putative proteins important for cell wall development

GO* terms	***S.cerevisae *genes**	**Homolgues in *T. rubrum***	**E value**	**Cluster**	**signal model**
**SW assembly (sensu Fungi)**	SPS19	DW703424	3.00E-16	IV	
	SPS19	DW691089	4.00E-10	IV	
	CDC10	DW693198	2.00E-67	IV	septation machinery;
	CDC10	DW698270	2.00E-17	IV	septation machinery;
**spore wall (sensu Fungi)**	CDC11	DW699003	4.00E-24	IV	septation machinery;
**signal transduction**	SLT2	DW703091	1.00E-112	IV	MAPK pathways
	BCY1	DW704384	1.00E-66	V	cAMP/PKA pathways
**CW organization and biogenesis**	SSD1	DW695092	8.00E-58	I	
	SLT2	DW703091	1.00E-112	IV	septation machinery;
	SLA2	DW686949	1.00E-26	II	MAPK pathways
	GSC2	DW687269	0	I	cAMP/PKA pathways
	PSA1	DW707265	9.00E-24	IV	MAPK pathways
	PMI40	DW702805	1.00E-53	IV	MAPK pathways
**cell wall chitin biosynthesis**	TOS1	DW708648	2.00E-19	V	
**cell wall (sensu Fungi)**	TDH3	DW694548	1.00E-45	I	MAPK pathways
	TDH2	DW711028	2.00E-99	I	
	GAS4	DW703981	1.00E-48	IV	MAPK pathways
	DAN4	DW693640	4.00E-09	V	
	CRH1	DW687789	7.00E-14	I	

* Gene Ontology

**Table 4:** Classification of protein important for cell polarity development

**GO terms**	***S.cerevisae *Genes**	**Homolgues in *T.rubrum***	**E value**	**Clusters**	**Class**
**axial bud site selection**	PFY1	DW684580	1.00E-31	IV	actin cytoskeleton
**bipolar bud site selection**	SLA2	DW686949	1.00E-26	II	actin cytoskeleton
	PFY1	DW684580	1.00E-31	IV	actin cytoskeleton
	ACT1	DW685603	0	III	actin cytoskeleton; MAPK pathways
**bud site selection**	CDC10	DW698270	2.00E-17	IV	septation machinery; MAPK pathways
	CDC10	DW693198	2.00E-67	IV	septation machinery; MAPK pathways
**Establishment of polarity**	CDC11	DW699003	4.00E-24	IV	septation machinery; MAPK pathways
	MLC1	DW702550	3.00E-25	III	actin cytoskeleton
	TPM1	DW696767	7.00E-30	IV	actin cytoskeleton
	RDI1	DW708650	9.00E-44	III	Rho-GTPase modules
	BCY1	DW704384	1.00E-66	IV	cAMP/PKA signaling pathways
	MEP2	DW680730	1.00E-15	IV	
**Invasive growth**	RAS2	DW683586	5.00E-16	I	RAS-type GTPase modules
	RAS2	DW679797	2.00E-24	IV	RAS-type GTPase modules
**Pscudohyphal growth**	RAS2	DW680378	1.00E-25	IV	RAS-type GTPase modules

* Gene Ontology
