# Supplementary material for: The use of global transcriptional analysis to reveal the biological and cellular events involved in distinct development phases of Trichophyton rubrum conidial germination
Source: BMC Genomics. 2007 Apr 11;8:100. doi: 10.1186/1471-2164-8-100 (PMC1871584; doi:10.1186/1471-2164-8-100)
Supplement: Additional file 1 — The data provided statistical analysis results for verifying the statistical significance between clusters of selected genes and real time PCR verification for microarray data. [file 1471-2164-8-100-S1.doc]

**1. The statistical analysis result for verifying the statistical significance between clusters.**

Oneway

The first column “1, 2 3, 4, 5” corresponding to the cluster I-V, respectively.

Post Hoc Tests

The first column “1, 2 3, 4, 5” corresponding to the cluster I-V, respectively.

**2 Validation of microarray data by real-time RT-PCR.**

In order to verify the microarray result, the relative expression levels of 8 genes at several time points (0, 4, 10, and 15) were estimated by Quantitative real-time RT-PCR. Gene-specific primers were designed for the genes of interest and the 18S rRNA using Primer Express software (Applied Biosystems) and are shown in Table 1.

**Table 1** Gene-specific primers used for real-time RT-PCR assays

| **Target** | **Primer Sequence*^a^*** |
| --- | --- |
| 18S rRNA | F,5'- CGCTGGCTTCTTAGAGGGACTAT -3' |
|  | R,5'- TGCCTCAAACTTCCATCGACTT -3' |
| DW679821 | F,5’- GAGGTGTTTATCTTTTCGCTGTC --3’ |
|  | R,5’- AGGTTTGTATTTGGGGTATCC --3’ |
| DW694001 | F,5’- ATCAAGGAACAGAAGCAACG --3’ |
|  | R,5’- TGGAAGGTGGGCAGAGTAA --3’ |
| DW698978 | F,5’- CCCATCCCGAGTTATTTCC --3’ |
|  | R,5’- TTTACCCATACGCTTCATCAG --3’ |
| DW691154 | F,5’- AACCTGACGAGCAAACCAA --3’ |
|  | R,5’- AATGACAACAGAGGCGATAAAG --3’ |
| DW699406 | F,5’- CCTTTTAGGTTCCCGCTGAG --3’ |
|  | R,5’- GGCAAATAACAACAACGCAAG --3’ |
| DW699957 | F, 5’- AAGTTTCCGCCAATGCCA --3’ |
|  | R,5’- ACCCTTCAATGCGTCCAG --3’ |
| DW699255 | F, 5’- CCCACCAGAATAACAGATGC --3’ |
|  | R,5’- CAGTGATGTGAACTCCGAGCT --3’ |
| DW700354 | F, 5’- GAGACGCCGATAAGGCAGAC --3’ |
|  | R,5’- TTCAGGGCAGGTGGTAAGC --3’ |
| DW702524 | F, 5’-TACCTTTGCCTTTGTCTGCC --3’ |
|  | F, 5’-TAATCTGGGTGCCGTTGC --3’ |
| DW683219 | F, 5’-ATCAAGGAACAGAAGCAACG --3’ |
|  | F, 5’- GGAAGGTGGGCAGAGTAACA --3’ |

*^a^* F, forward; R, reverse.

The PCR cycle consisted of AmpliTaq Gold activation at 95°C for 10 min, followed by 40 cycles of denaturation at 95°C for 15 s and annealing/extension at 58°C for 1 min. A dissociation curve was generated at the end of each PCR cycle to verify that a single product was amplified using software provided with the 7000 Sequence Detection System. The changes in fluorescence of SYBR Green I dye in each cycle were monitored by the system software, and the calculated threshold cycle (*C*_t_) for each gene amplification was normalized to *C*_t_ of the 18S rRNA gene amplified from the corresponding sample before calculating the fold change from a selected time point to 0 time point using the following formula:

fold change = 2–
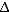

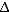
C_t_

where
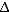

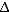
*C*_t_ for gene *j* = (*C*_t_,*_j_* – *C*_t,18S rRNA_)_a time point_ – (*C*_t_,*_j_* – *C*_t,18S rRNA_)_time point 0_.

The real-time RT-PCR assays results were showed in

**Table 2. The relative fold change for 10 genes listed in Table 1 determined by quantitative real-time RT-PCR and microarray hybridization results**.

| ESTs | Cluster | 0 hr | | 4 hr | | 10hr | | 15r | | *r* |
| --- | --- | --- | --- | --- | --- | --- | --- | --- | --- | --- |
|  |  | R^a^ | M^b^ | R^a^ | M^b^ | R^a^ | M^b^ | R^a^ | M^b^ |  |
| DW679821 | I | 1 | 1 | 0.458 | 0.4827956 | 0.393 | 0.3528672 | 0.325 | 0.3337636 | 0.996 |
| DW694001 | IV | 1 | 1 | 1.648 | 3.331617 | 1.516 | 4.7668537 | 2.66 | 5.7810314 | 0.859 |
| DW698978 | IV | 1 | 1 | 3.817 | 2.8971517 | 4.082 | 3.1678551 | 3.287 | 2.9819751 | 0.979 |
| DW691154 | III | 1 | 1 | 7.571 | 2.4231938 | 17.239 | 18.971541 | 18.647 | 22.226249 | 0.962 |
| DW699406 | III | 1 | 1 | 0.858 | 1.223877 | 1.877 | 2.0662205 | 1.495 | 2.4574132 | 0.807 |
| DW699957 | V | 1 | 1 | 16.851 | 3.6974249 | 9.624 | 3.0518661 | 9.515 | 3.6493771 | 0.892 |
| DW702524 | II | 1 | 1 | 1.472 | 1.1364942 | 1.327 | 1.3442048 | 2.008 | 1.6315185 | 0.898 |
| DW683219 | II | 1 | 1 | 2.65 | 1.65402885 | 4.125 | 1.90301182 | 2.396 | 1.981882 | 0.792 |
| DW699255 | V | 1 | 1 | 2.183 | 3.1367872 | 1.452 | 1.8660252 | 1.823 | 1.8301019 | 0.93 |
| DW700354 | V | 1 | 1 | 4.304 | 4.1661408 | 3.135 | 2.7891405 | 4.093 | 2.5168176 | 0.879 |

^a^ Column R is the fold change relative to time point 0 hr determined by quantitative real-time RT-PCR; ^b^ Column M is the fold change relative to time point 0 hr determined by microarray hybridization results.

The data in Table2 showed that there was a strong positive correlation (r> 0.79) between the two techniques.
